# Sonoactivated polycrystalline Ni electrodes for alkaline oxygen evolution reaction

**DOI:** 10.1016/j.ultsonch.2022.106013

**Published:** 2022-04-23

**Authors:** Faranak Foroughi, Alaa Y. Faid, Svein Sunde, Bruno G. Pollet

**Affiliations:** a Hydrogen Energy and Sonochemistry Research Group, Department of Energy and Process Engineering, Faculty of Engineering, Norwegian University of Science and Technology (NTNU), NO-7491 Trondheim, Norway; b Electrochemistry Research Group, Department of Materials Science and Engineering, Faculty of Natural Sciences. Norwegian University of Science and Technology (NTNU), NO-7491 Trondheim, Norway; c Green Hydrogen Lab (GH2Lab), Pollet Research Group, Hydrogen Research Institute, Université du Québec à Trois-Rivières, 3351 Boulevard des Forges, Trois-Rivières, Québec G9A 5H7, Canada

**Keywords:** Electrolysis, Oxygen evolution reaction, Alkaline, Nickel, Ultrasound

## Abstract

The development of cost-effective and active water-splitting electrocatalysts is an essential step toward the realization of sustainable energy. Its success requires an intensive improvement in the kinetics of the anodic half-reaction of the oxygen evolution reaction (OER), which determines the overall system efficiency to a large extent. In this work, we designed a facile and one-route strategy to activate the surface of metallic nickel (Ni) for the OER in alkaline media by ultrasound (24 kHz, 44 W, 60% acoustic amplitude, ultrasonic horn). Sonoactivated Ni showed enhanced OER activity with a much lower potential at + 10 mA cm^−2^ of + 1.594 V *vs.* RHE after 30 min ultrasonic treatment compared to + 1.617 V *vs.* RHE before ultrasonication. In addition, lower charge transfer resistance of 11.1 Ω was observed for sonoactivated Ni as compared to 98.5 Ω for non-sonoactivated Ni. In our conditions, ultrasound did not greatly affect the electrochemical surface area (*A*_ecsa_) and Tafel slopes however, the enhancement of OER activity can be due to the formation of free OH^•^ radicals resulting from cavitation bubbles collapsing at the electrode/electrolyte interface.

## Highlights

• Ultrasound treatment was used as an activation route to enhance the OER activity of polycrystalline Ni.
• Sonoactivated polycrystalline Ni electrode showed lower overpotential and lower charge transfer resistance towards OER.
• Ultrasound treatment did not significantly affect the electrochemical surface area of polycrystalline Ni.

## 1 Introduction

**Fig. 1:**
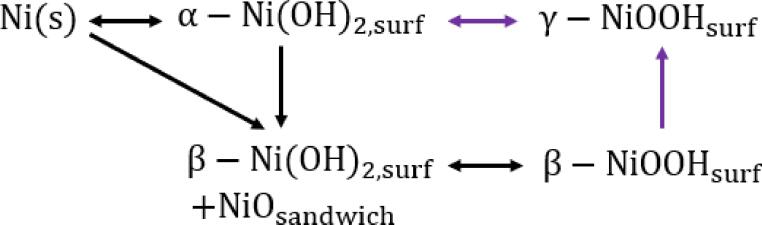
Schematic diagram of relationships between metallic Ni and various O containing surface compounds formed during anodic oxidation of polycrystalline Ni in aqueous alkaline media [4].

**Fig. 2:**
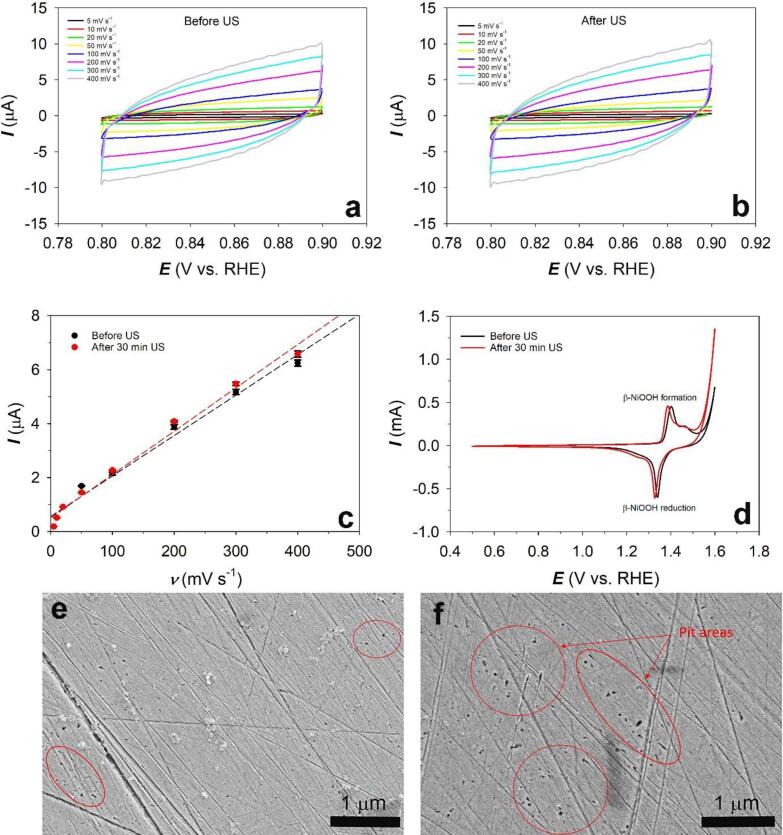
Cyclic voltammetry (CV) scans of the Ni(poly) electrode in 1.0 M aqueous KOH solution at different scan rates (5, 10, 20, 50, 100, 200, 300 and 400 mV s^−1^), +0.80 ≤ *E*_app_ ≤ +0.90 V *vs.* RHE and *T* = 298 K (a) before and (b) after ultrasonication for 30 min, (c) The capacitance method: plots of current *vs*. scan rate as well as linear regressions of each data set (dotted lines), obtained from the CV experiments at an applied potential of + 0.85 V *vs.* RHE before and after 30 min ultrasonication, (d) CV profiles of Ni(poly) at + 0.5 ≤ *E*_app_ ≤ +1.60 V *vs.* RHE and a scan rate of *ν* = 100 mV s^−1^ before and after 30 min US. SEM images of the Ni(poly) electrode (e) before and (f) after 30 min ultrasonication.

The water electrolysis process occurs through two simultaneous half-cell reactions: the oxygen evolution reaction (OER) on the anode and the hydrogen evolution reaction (HER) on the cathode. The Alkaline OER is a 4-electron–proton transfer process that makes the reaction sluggish with high overpotential and complex reaction mechanisms [1], [2]. Nickel (Ni)-based compounds including Ni-based oxides and (oxy)hydroxides are among the most efficient precious-metal-free catalysts for alkaline OER due to their desirable advantages such as enhanced reaction kinetics and structure/performance stability [3]. Relationships between metallic Ni and various O-containing surface compounds formed during anodic oxidation of polycrystalline Ni in aqueous alkaline media can be described by the Bode diagram (Fig. 1) [4]. Mild anodic polarization of metallic Ni results in the reversible formation of α-Ni(OH)_2_; moderate anodic polarization results in the irreversible conversion of α-Ni(OH)_2_ into β-Ni(OH)_2_ as well as in the direct oxidation of Ni to β-Ni(OH)_2_; and, this process is accompanied by the development of NiO that is sandwiched between Ni and β-Ni(OH)_2_ (marked as a NiO sandwich in Fig. 2). The purple lines and the formation of γ-NiOOH were suggested by Bode [5]. The γ-NiOOH phase is believed to be the highest-achievable Ni oxidation state [6]. It is most commonly assumed that the β-NiOOH oxidation phase is most active towards the OER [7].

So far, many research efforts have focussed on improving the OER performance of Ni by the design and optimization of the catalyst structure [6], [8], [9].

Sonoelectrochemistry is the combination of ultrasound with electrochemistry. The use of ultrasound in electrochemistry offers many advantages including [10]: a) gas bubble removal at the electrode surface; b) solution degassing; c) disruption of the *Nernst* diffusion layer; d) enhancement of mass transport of electroactive specious through the double layer; and, e) activation and cleaning of the electrode surface. Recently, it was reported that ultrasonication greatly enhances the electrocatalytic properties of metallic surfaces [11], [12], [13], [14], [15], [16]. Our group also investigated the effect of ultrasound on Ni(poly) in alkaline media and found that the rate of the HER was greatly enhanced.

In this work, we investigated the effects of ultrasound (24 kHz) on the OER on polycrystalline Ni immersed in 1.0 M aqueous KOH solution at room temperature. We applied ultrasound (i) during linear sweep voltammetry (LSV) experiments and (ii) for surface treatment of the Ni(poly) electrode for 30 min and then we conducted the LSV experiments under *silent* conditions (in the absence of ultrasound).

## 2 Experimental

All electrochemical experiments were carried out using a potentiostat/galvanostat (BioLogic-SP 150) in a three-electrode configuration. The voltammetry experiments were performed using a double-jacketed sonoelectrochemical cell. Ultrasonication was applied by a *f* = 24 kHz ultrasonic probe (Hielscher UP200S, 200 W @ 60% fixed amplitude, the tip Ø = 14 mm, and the tip area = 153.9 mm^2^ (1.5386 cm^2^). The ultrasonic or acoustic power (*P*_acous_) was found to be 44 ± 1.40 W by calorimetrically using the methods of Margulis *et al.* [17] and Contamine *et al*. [18]. In order to keep the temperature at *T* = 298 ± 1 K a refrigerated circulator (JULABO, Germany) was connected to the sonoelectrochemical cell.

A polycrystalline nickel Ni(poly) disc (Ø = 5 mm) of geometric surface area (*A*_geom_) of 0.196 cm^2^ was used as a working electrode (WE). The WE was mechanically polished using alumina suspension (down to 0.05 μm, Buehler Micro polish) to obtain a mirror-like surface rinsed with UHP water, ultrasonicated in UHP water for ∼30 s and finally rinsed in UHP water under ultrasonic conditions. The reference electrode (RE) was a homemade reversible hydrogen electrode (RHE) [19]. All potential values in this work are reported with respect to the RHE. The counter electrode (CE) was a Ni mesh (40 mesh woven from 0.13 mm diameter wire, 99.99% metal basis, Alfa Aesar, Germany) in a rectangle shape (20.67 × 10.76 mm^2^). Its surface area was at least 10 times larger than that of the WE. The distance between the ultrasonic probe and the working electrode was ca. 3 cm. The experiments were carried out in N_2_ (g) (99.999%) saturated 1.00 M (pH = 13.7) aqueous KOH (Sigma-Aldrich, 99.99% in purity) solution prepared using ultra-high-purity water (Millipore, 18.2 MΩ cm in resistivity).

The performance of Ni(poly) towards the OER in aqueous alkaline electrolytes was investigated by a series of linear sweep voltammetry (LSV) in the potential region of + 1.10 ≤ *E*_app_ ≤ +1.70 V *vs.* RHE at the potential scan rate of *ν* = 0.30 mV s^−1^ in 1.0 M KOH aqueous solutions in the absence of ultrasound (*silent* conditions), during (with) ultrasound and after 30 min ultrasound.

The potential values from linear sweep voltammetry (LSV) experiments were *IR* corrected using the following equation (1):

(1) $$E_{\mathrm{IR}-corrected}=E_{\mathrm{app}}--IR$$

where *I* is the measured current and *R* is the electrolyte resistance, measured for each electrolyte employed. The *R* value was determined by electrochemical impedance spectroscopy (EIS) in the high-frequency region from the value of the *real* impedance (*Z^’^*) where the *imaginary* impedance (*Z*^’’^) is zero in the Nyquist plot. The EIS experiments were carried out in the 100 kHz to 0.1 Hz frequency (*f*) range with a voltage perturbation of + 10 mV at an applied potential of +1.60 V *vs.* RHE at *T* = 298 K.

The surface structure and morphology of the Ni(poly) electrodes before and after ultrasound treatment were studied using a scanning electron microscope (SEM) Zeiss-Ultra 55-FEG-SEM operating at 10 kV accelerating voltage.

## 3 Result and discussion

### 3.1 Study of the effect of ultrasound on the electrochemical surface area of polycrystalline Ni

In order to study the effects of power ultrasound on the electrochemical surface area of Ni(poly), the “capacitance” and “β-NiOOH” methods were used. The “capacitance” method consists of cycling the Ni electrodes at different scan rates in a non-faradic charging process to determine the electrochemical surface area (*A*_ecsa_) [20]. A series of cyclic voltammograms (CVs) on Ni(poly) in 1.0 M KOH were generated at different scan rates (5, 10, 20, 50, 100, 200, 300, 400 mV s^−1^) in the potential region of + 0.80 V *vs.* RHE to + 0.90 V *vs.* RHE. The double-layer capacitance value (*C*_dl_) was obtained by plotting the charging current (*I*_c_, A) *vs.* scan rate (*ν*, V s^−1^) and by using equation (2):

(2) $$\mathrm{Slope}=C_{\mathrm{dl}}=\frac{\Delta I_{c}}{\Delta v}$$

The electrochemical surface area was calculated by using the specific capacitance density (c) of 40 μF cm^−2^ and equation (3) [20], [21].

(3) $$A_{\mathrm{ecsa}}=\frac{C_{\mathrm{dl}}}{C}$$

Fig. 2a and 2b show the CVs of the Ni(poly) electrode before and after 30 min of ultrasonication at different scan rates (5, 10, 20, 50, 100, 200, 300, and 400 mV s^−1^) in the potential range of + 0.80 ≤ *E*_app_ ≤ +0.90 V *vs.* RHE where non-faradic currents occur. Fig. 2c shows plots of current *vs.* scan rate at a potential of + 0.85 V *vs.* RHE before and after 30 mins of ultrasonic exposure.

The “β-NiOOH” method consisted of integrating the β-NiOOH reduction peak once steady-state polarization was reached at a high scan rate. The β-NiOOH method was carried out by running 10 CV cycles from + 0.50 ≤ *E*_app_ ≤ +1.60 V *vs*. RHE at a scan rate of *ν* = 100 mV s^−1^ before and after 30 min US (Fig. 2d). The *A*_ecsa_ values for this method were calculated using the β-NiOOH reduction peak of the 10th cycle (from 1.2 to 1.4 V *vs.* RHE) divided by the specific charge density of 420 μC cm^−2^ (equation (4)) [20].

(4) $$A_{ecsa}=\frac{Q}{420}$$

where *Q* is the charge associated with the β-NiOOH reduction peak. The *A*_ecsa_ values before and after 30 min of ultrasonication treatment for both capacitance and beta methods are summarised in Table 1. It needs to be mentioned that the difference between the *A*_ecsa_ values from the “capacitance” and the “β-NiOOH” methods is related to the basis of measurements of both methods. The capacitance method is related to conductivity and homogeneity of surface for double layer charging while the beta method is related to the faradaic reaction of nickel hydroxide to nickel oxyhydroxide transformation [20]. It can be observed from Table 1 that ultrasound does not seem to affect the electrochemical surface area of the Ni(poly) electrode, indicating that the electrochemical surface area was not significantly modified due to erosion caused by the implosion of acoustic cavitation bubbles on the electrode surface [22]. Fig. 2e and 2f show the SEM images of Ni(poly) before and after 30 min US. Before US a smooth surface is seen except the scratches due to mechanical polishing. After 30 min US some irregular pits could be observed, however, it is unclear whether these arose from the actions of inter-facial ultrasound. Such features are sometimes found widely scattered across non-sonicated surfaces (see, for example, some pits in non-sonicated electrode Fig. 2e). The pit areas in both non-sonicated and sonicated electrodes have been marked red in Fig. 2e and 2f. These pits have little influence on electrochemical measurements because there are very few of them and their contribution to total *A*_ecsa_ is relatively small. Aqueous ultrasonication did not significantly roughen the electrode and the surface roughness remained almost unchanged [23].

**Table 1:** The electrochemical surface area (*A*_ecsa_) of the Ni(poly) electrode before and after 30 min ultrasonication in 1.0 M aqueous KOH solution and *T* = 298 K. (n = 3).

Material	*A*_ecsa(capacitance)_ (cm^2^)	*A*_ecsa (beta)_ (cm^2^)
Ni (before ultrasonication)	0.38 ± 0.009	0.88 ± 0.004
Ni (after ultrasonication)	0.40 ± 0.005	0.95 ± 0.035

### 3.2 Study of the effect of ultrasonic power on the oxygen evolution reaction

**Fig. 3:**
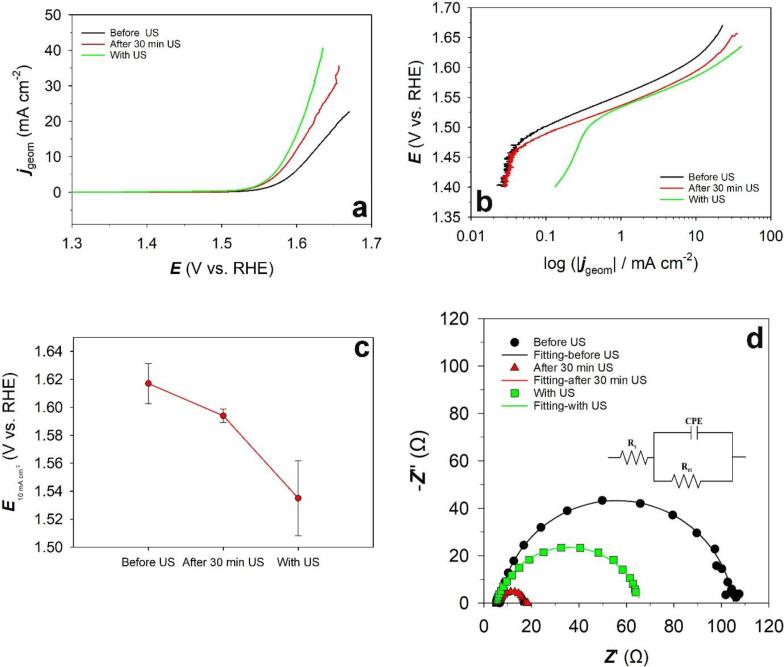
(a) Linear sweep voltammograms (LSVs) for the OER, (b) overlaid Tafel plots (c) plot of *E* at 10 mA cm^−2^*vs.* various US conditions and (d) Nyquist plots; Inset shows the equivalent circuit used to fit the impedance data of the Ni(poly) electrode in N_2_ saturated 1.0 M aqueous KOH solution at a scan rate of *ν* = 0.3 mV s^−1^ and at *T* = 298 K before US, with US and after 30 min US treatment.

The effect of ultrasound on the oxygen evolution reaction (OER) at Ni(poly) in 1.0 M aqueous KOH solution was investigated by linear sweep voltammetry (LSV). Fig. 3a shows the LSVs for the OER on Ni(poly) in N_2_ saturated 1.0 M KOH aqueous solutions at a low scan rate of *ν* = 0.3 mV s^−1^ before, during (with) and after 30 min ultrasonic treatment. It can be observed that the ultrasonic (US) treatment increases the OER activity. Fig. 3b demonstrates the Tafel plots obtained from the LSV curves in the OER region. Tafel slopes (*b*) at low and high overpotentials and the potential at + 10 mA cm^−2^ (*E*_+10 mA cm_^-2^) are tabulated in Table 2. Results from Table 2 indicate that lower potential requires to reach + 10 mA cm^−2^ in presence of ultrasound and after ultrasonic treatment. However, even when ultrasound is “on” during the OER experiments, the lower overpotential at + 10 mA cm^−2^ is required when compared to after ultrasonic treatment.

**Table 2:** Tafel slopes (*b*) and potential at + 10 mA cm^−2^ (*E*_10 mA cm_^-2^) for the OER on Ni(poly) electrode in 1.0 M KOH aqueous solutions before US, with US and after 30 min US.

Ni(poly)	b* (mV dec^-1^) at low overpotential	b** (mV dec^-1^) at high overpotential	*E _+_*_10 mA cm_^-2^ (V *vs*. RHE)
Before US	52	141	1.617
With US	55	90	1.535
After 30 min US	50	130	1.594

* 1.45 ≤ *E* ≤ 1.55.

** 1.60 ≤ *E* ≤ 1.65.

Ni-based materials show the Tafel slope values between 40 mV dec^-1^ to 130 mV dec^-1^. Also, it is well known that there are generally two Tafel regions for the OER, separated at ∼ 1.5 V *vs.* RHE in 1.0 M KOH [6], [7]. According to Table 2, the Tafel slopes of 52, 55, 50 mV dec^-1^ at low overpotentials and 141, 90 and 130 mV dec^-1^ at high overpotentials were obtained for the OER on Ni(poly) before ultrasonication (US), with US and after 30 min US, respectively. The Tafel slopes are in good agreement with the literature [7], [24], [25]. By comparing the Tafel slopes under different US conditions reported in Table 2, it can be concluded that ultrasound does not change the Tafel slopes significantly for the OER and does not affect the mechanism of OER. It is worth mentioning that the experiments have been repeated several times and almost the same values have been obtained showing the reproducibility of the work.

Fig. 3c illustrates the plot of *E* at + 10 mA cm^−2^ (*E_+_*_10 mA cm_^-2^) *vs.* different ultrasonic conditions. It can be seen in Fig. 3c that the overpotential to reach + 10 mA cm^−2^ decreases when US is “on” during the OER experiment.

Fig. 3d shows the Nyquist representation of the impedance data of Ni(poly) before US, with US and after 30 min US at *T* = 298 K and *E* = +1.60 V *vs.* RHE. For all US conditions, a depressed semi-circle can be seen. Accordingly, the data were approximated by the modified Randles circuit shown in Fig. 3d, whereas the capacitance is replaced by a constant phase element. Note, for *α* = 1 the CPE reflects an ideal capacitance. *R*_s_ correlates with the cell ohmic resistance (electrodes). *R*_ct_ represents the charge transfer resistance and may also include other contributions such as the adsorption of intermediates. CPE is a constant phase element that is often associated with the capacitive charging of a rough electrode. The parameters obtained from the EIS measurement are shown in Table 3. According to Table 3, the Ni(poly) electrode after 30 min US treatment has the lowest charge transfer resistance compared to the two other conditions. While the *R*_s_ are almost constant in all US conditions. Since no significant increase in the electrochemical surface has been observed on Ni(poly) by applying US, the enhancement of OER activity of Ni(poly) after ultrasonication treatment could be due to the reaction of radicals at the electrode/electrolyte interface such as (OH^•^, H^•^, H_2_O_2_, etc) caused by collapsing cavitation bubbles. It was reported before that such radicals could react with the electrolyte species and produce a secondary sonochemical reaction [15], [16], [26], [27].

**Table 3:** Cell ohmic resistance (*R*_s_) and charge transfer resistance (*R*_ct_).

Ni(poly)	*R_s_* (Ω)	*R_ct_* (Ω)
Before US	6.69	98.5
With US	6.60	61.0
After 30 min US	6.25	11.1

## 4 Conclusions

We have developed a simple *in-situ* method to activate Ni(poly) electrodes in 1.0 M aqueous KOH solution towards the OER by ultrasonic treatment (24 kHz, 60% amplitude, 44 W) for 30 min. It was shown that ultrasound improves Ni(poly) OER activity by reducing the overpotential needed to achieve + 10 mA cm^−2^ by –23 mV and charge transfer resistance from 98.5 Ω before US to 11.1 Ω after 30 min US treatment. However, the US treatment does not affect the electrochemical surface area of Ni(poly) or Tafel slope. The enhancement of OER activity of Ni(poly) could be attributed to the formation of free radicals by collapsing cavitation bubbles and the secondary sonochemical reactions at the electrode/electrolyte interface. However, understanding the exact reason and the mechanism will still need a wide range of experiments and spectroscopy measurements.

## Declaration of Competing Interest

The authors declare that they have no known competing financial interests or personal relationships that could have appeared to influence the work reported in this paper.
